# More Effort, Less Fatigue: The Role of Interest in Increasing Effort and Reducing Mental Fatigue

**DOI:** 10.3389/fpsyg.2021.755858

**Published:** 2021-11-19

**Authors:** Marina Milyavskaya, Brian M. Galla, Michael Inzlicht, Angela L. Duckworth

**Affiliations:** ^1^Department of Psychology, Carleton University, Ottawa, ON, Canada; ^2^School of Education, University of Pittsburgh, Pittsburgh, PA, United States; ^3^Department of Psychology, University of Toronto, Toronto, ON, Canada; ^4^Department of Psychology, University of Pennsylvania, Philadelphia, PA, United States

**Keywords:** cognitive work, effort, interest, self-efficacy, fatigue

## Abstract

People generally prefer easier over more difficult mental tasks. Using two different adaptations of a demand selection task, we show that interest can influence this effect, such that participants choose options with a higher cognitive workload. Interest was also associated with lower feelings of fatigue. In two studies, participants (*N* = 63 and *N* = 158) repeatedly made a choice between completing a difficult or easy math problem. Results show that liking math predicts choosing more difficult (vs. easy) math problems (even after controlling for perceived math skill). Two additional studies used the Academic Diligence Task (Galla et al., 2014), where high school students (*N* = 447 and *N* = 884) could toggle between a math task and playing a video game/watching videos. In these studies, we again find that math interest relates to greater proportion of time spent on the math problems. Three of these four studies also examined perceived fatigue, finding that interest relates to lower fatigue. An internal meta-analysis of the four studies finds a small but robust effect of interest on both the willingness to exert greater effort and the experience of less fatigue (despite engaging in more effort).

## Introduction

People go through their lives making choices both large and small. Many of these choices involve the decision to expend or conserve effort: Do I pack a lunch or buy one? Do I reread my notes to prepare for an exam or study by testing myself on the material? Do I watch TV or work on Sudoku puzzles in my spare time? Typically, people prefer to conserve effort and take the easier route (Hull, 1943; Kool et al., 2010). However, they sometimes choose to engage in more effortful activities: Sudoku instead of TV, more difficult courses instead of “easy As,” and cognitively demanding video games. Here, we examine why people engage in effortful activity in the absence of external or immediate rewards and contrast interest and self-efficacy as two possible sources of value that are inherent to the activity itself. We also investigate whether interest and self-efficacy relate to the phenomenology of effort. Ultimately, we wonder if feelings of effort are not merely related to how demanding or difficult a task is, but also the product of how interested and efficacious a person feels while performing the task.

### Cognitive Effort

Effort can be defined as “the intensification of mental or physical activity” (Inzlicht et al., 2018), frequently in response to task demands, and used in the service of a goal. Researchers have proposed that cognitive effort feels aversive (Botvinick, 2007; Inzlicht et al., 2015; Saunders et al., 2015), and that people are “cognitive misers” who use heuristics in order to limit the effort used in making decisions (Fiske and Taylor, 1991). In support of this, past studies (e.g., Kool et al., 2010; Westbrook et al., 2013; Dunn et al., 2016) find that when given a series of choices, participants choose the less effortful option on the vast majority of trials. Additionally, effort tracks closely with the likelihood of error, such that error-prone but brief tasks are considered more effortful than easier tasks performed for a longer duration (Dunn et al., 2019).

Given that effort is costly and undesirable, valuation theories propose that rewards are required to compensate for the cost of effort. These rewards are typically assumed to be external, with most research using monetary rewards (e.g., Hartmann et al., 2013; Westbrook et al., 2013). However, in many cognitively demanding activities (e.g., crossword puzzles, Sudoku, etc.), external rewards are typically absent or irrelevant—what *is* evident are intrinsic incentives, such as positive affect (Woolley and Fishbach, 2015). Similarly, feelings of self-efficacy are also sometimes acknowledged as rewarding (Bandura, 1982). That is, people are thought to engage in effort for internal rewards, such as the subjective experience of efficacy (Satterthwaite et al., 2012), competence (Deci and Ryan, 2000), or self-esteem/self-definition (Gendolla and Richter, 2010). Importantly, inherent interest and enjoyment of the task itself may serve a similar rewarding function to the extrinsic rewards that spur individuals to exert effort (see also Ainslie, 2013; Galla et al., 2018).

This idea that effort can be intrinsically valued is at odds with the argument that effort is *inherently* aversive. Indeed, a recent review argues that effort is sometimes paradoxically valuable (Inzlicht et al., 2018), while other research finds that perceptions of effort are subjectively determined (Dunn et al., 2016). In the present paper, we propose that defining something as effortful “cognitive work” has less to do with objective criteria (e.g., the cognitive operations involved) and more to do with the appraisal of the task in terms of personal interest.

### Interest and Self-Efficacy

Interest has been examined both as an emotion (Silvia, 2008) and as “an individual predisposition to attend to certain objects and events and to engage in certain activities” (Ainley et al., 2002). Interest as an individual difference is thought to interact with situational cues to elicit situational interest, described as a psychological state or emotion, whose function is “to motivate learning and exploration” (Silvia, 2008). Personal interest inherent in a task has long been described by self-determination theory as intrinsic motivation—doing something for its own sake, rather than for external or internal rewards or consequences (Deci and Ryan, 2000). Indeed, intrinsic motivation is frequently operationalized as persistence on a challenging task during a free-choice period (Deci, 1971)—essentially, a greater willingness to exert effort. Further, interest can lead people to exert greater effort and persist longer in subsequent effortful tasks than mere positive affect (Thoman et al., 2011), pointing to interest’s unique experiential nature. Other research suggests that engaging in cognitive effort can be a pleasant experience, pursued for its own sake (Csikszentmihalyi and LeFevre, 1989; Waterman, 2005); we propose that interest may spur such engagement.

When people persist on a difficult task in the absence of external rewards, another possible explanation is that they are driven by feelings of self-efficacy derived from successful engagement or completion of a task. For example, the satisfaction one gets from completing a crossword puzzle (rather than the enjoyment of the process of working on the puzzle) could explain why people are willing to expend effort on this pursuit. In these cases, prior experiences with success at similar tasks could lead people to expect further success in the future; people who are good at certain types of tasks will likely experience feelings of competence from engaging in them. Indeed, feelings of predicted self-efficacy for a new task could be expected to lead to greater willingness to exert effort in pursuing the task (Bandura, 1977). Similarly, feelings of self-efficacy might lead to increased engagement in academic tasks (e.g., Liem et al., 2008).

Although self-efficacy is related to intrinsic motivation (that is, people generally enjoy and are interested in tasks where they feel efficacious), these concepts are distinct. For example, someone might enjoy working on crossword puzzles despite not feeling confident in their abilities. Alternatively, a person may excel at math yet not enjoy or be particularly interested in the subject. Indeed, in studies where both are assessed, the relation between interest and self-efficacy is typically moderate (Rottinghaus et al., 2003). Additionally, past research found that interest (in an academic subject) predicts academic self-regulation even after controlling for self-efficacy (Lee et al., 2014). In the present studies, we similarly sought to distinguish inherent interest from self-efficacy, and explore their separate influences on the willingness to exert more effort in a given task. Specifically, we expected that interest would be related to greater effort, even after controlling for self-efficacy^1^.

### Phenomenology of Fatigue

In addition to actual behavior, we are also interested in the phenomenology of effort. That is, how do people *feel* when they engage in effortful (rather than easy) tasks? Previous research has found that cognitive effort is related to negative affect (Saunders et al., 2015) and its physiological correlates (Elkins-Brown et al., 2016). Here, we are particularly interested in subjective feelings of fatigue. Indeed, fatigue has been conceptualized as a negative affective state that arises when a person exerts effort on a given task when they would prefer to disengage and shift to an alternative (Hockey, 2013). It is frequently found in situations requiring sustained cognitive effort or attention, and typically leads to task disengagement (see Kurzban et al., 2013; Kurzban, 2016).

One question that then arises is whether this fatigue is based on the task itself. Some research suggests that a person’s actual exertion of effort and their perceived exertion of effort (and subsequent feelings of fatigue) are only loosely related. For example, a recent paper that contrasted effort (mentally manipulating a four-digit number) with boredom (passively observing strings of numbers) found that participants in the boredom condition reported significantly more fatigue, despite reporting less effort (Milyavskaya et al., 2019). In another study, people who performed an easy task, but were then told that the task was depleting, subsequently reported feeling depleted and acted accordingly (Clarkson et al., 2010). These studies further support the proposition that exerting effort does not always lead to subjective feelings of fatigue.

One possible reason for such mismatches between effort and subjective fatigue is that sometimes exerting effort does not feel effortful. In line with others (e.g., Clarkson et al., 2010; Job et al., 2010; Francis and Job, 2018), we suggest that fatigue is a matter of perception. Importantly, we propose that people will not *feel* fatigued when they are engaged in an interesting or enjoyable activity, even if it is objectively demanding. This is consistent with prior research on interest and autonomous motivation more generally (including personal value and fit with core values; as distinguished from controlled motivation, such as doing something for external rewards or because of guilt or shame), which has shown that autonomous motivation can reduce feelings of fatigue and increase vitality (Ryan and Deci, 2008). Importantly, studies have found that pursuing an activity for autonomous reasons makes it *feel* less effortful or depleting than when the same activity is pursued for non-autonomous reasons, and that people experience less temptations that interfere with such activities. For example, Muraven (2008) found that people who exert self-control for autonomous reasons do not experience the depletion effect—that is, they persisted longer on an effortful handgrip task after initially exerting effort in resisting the temptation to eat cookies. Extending these findings to a longer time frame, Werner et al. (2016) found that pursuing more autonomous goals across a semester led to these goals being perceived as less effortful (compared to a person’s other goals). And in an experience sampling study, autonomous motivation was related to experiencing fewer temptations (Milyavskaya et al., 2015), which in turn was related to lower perceptions of depletion (Milyavskaya and Inzlicht, 2017). Testing these predictions in a task that tracks effort and feelings of depletion in real time, we expect that people who enjoy an activity will feel less tempted by attractive alternatives and less fatigued even after exerting effort.

### Present Studies

Based on the past research reviewed above, the current set of studies tests whether interest leads to greater use of mental effort along with reduced feelings of fatigue. We also contrast the effects of interest with the effects of self-efficacy. The present paper is a merging of two separate research enterprises by two separate labs, once we realized that we both had data to examine similar phenomena. The studies presented herein are thus very different—two studies investigate the phenomenon online or in the lab, using undergraduate students, while two others do so in field studies in high schools with adolescent participants. In all four studies, participants have a recurring choice between engaging in less or more effortful behavior. In the two lab studies, the choice is between a difficult and easier task; in the field studies, the choice is between a math task and playing a video game/watching videos. In both sets of studies, one choice requires more effort than the other. We are interested in both the proportion of time that participants spend engaging in the more effortful behavior, and in participants’ reported feelings of fatigue after engaging in these behaviors. Across all studies, we investigated whether greater interest/enjoyment of the subject matter would be related to spending more time on the effortful options, and also to feeling less fatigued following the use of effort. We initially expected interest/enjoyment to play a role independent of self-efficacy^2^. Across all studies, we report how we determined our sample size and all data exclusions. All materials for each study and any alternative analyses (described in the text or footnotes) are available on the open science framework (OSF) at https://osf.io/sn376.

## Study 1

In this study, we examined people’s choices in a demand selection task (Kool et al., 2010), in which participants repeatedly chose between an easy (*add1*) or effortful (*add3*) versions of a number-manipulation task. In both versions, participants briefly saw four digits appear on a computer screen and had to add either one (the *add1* version) or three (the *add3* version) to each digit while holding these digits in memory, and then enter the new number into the program. Although both tasks used basic math (adding either one or three to another single-digit number), previous research has found that the *add3* task is more cognitively demanding than the *add1* task (Kahneman et al., 1969). In each trial, participants chose which of the two tasks they would attempt; they completed multiple trials over the course of 15 min. At the end, participants reported on feelings of fatigue and completed questionnaires, including one item assessing how much they generally like math and another assessing how good they believe they are at math (see^3^ for all materials and the data). We hypothesized that students who generally enjoy math would be willing to work harder at math-related problems, and would thus select more *add3* problems than those who did not like math. Importantly, since we wanted to rule out self-efficacy as the explanation, we expected that math interest, but not necessarily math self-efficacy, would predict choosing more *add3* problems. In addition, we hypothesized that math interest would predict perception of less fatigue despite selecting more *add3* problems.

### Participants and Procedure

Participants were 83 undergraduate students at the University of Toronto Scarborough, who completed a multi-trial online study on working memory for course credit^4^. Using Inquisit software, participants first completed five practice trials each of the *add3* and *add1* tasks,^5^ and then completed the chosen task for 15 min, involving repeated choices between the *add1* and *add3*. Because everyone completed 15 min of the task, the number of trials varied per person (depending on how quickly they completed each trial). Following the task, they completed a series of questionnaires (see full list on OSF), including a 2-item measure of fatigue (“How fatigued are you right now?”; “How mentally exhausted do you feel right now?”, both rated on a scale of 1 (*not at all*) to 10 (*very much*), *r* = 0.61, *p* < 0.001), as well as one question about how much they liked math (from 1 = *I really dislike math* to 5 = *I really like math*) to assess interest and one about how good they believed they were at math (1 = *I’m really good at math*; 5 = *I’m really bad at math*) to assess self-efficacy. The item referring to being good at math was reverse-coded so that higher numbers indicated beliefs of being better at math (i.e., higher self-efficacy). Nineteen participants who did not correctly complete a single *add1* or the *add3* practice problems were removed (since it was clear that they were not paying attention and/or did not understand the task), as well as one participant who did not complete the follow-up questionnaire, resulting in a final sample size of 63.

#### Add1/Add3 Choice Task

**FIGURE 1:**
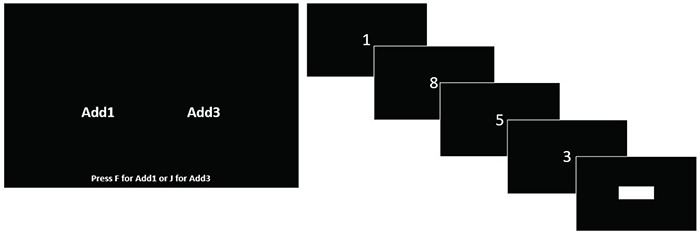
Add1/add3 choice task used in Studies 1 and 2. Participants saw the choice screen, and once they made a selection, were presented with four numbers and a box to enter the response.

In this task, participants were asked to indicate in each trial whether they wanted to do an *add1* or *add3* problem in that trial. In each trial, participants first saw the choice screen (see Figure 1), and pressed F to do an *add1* problem, or J to do an *add3* problem. After their selection, a circle appeared in the center of the screen, followed by four randomly selected digits presented one at a time, in intervals of 900 ms. After four digits, another circle appeared for 900 ms, followed by a response box presented on screen for up to 4 s. For the *add1* problems, participants had to add one to each digit and enter the response in the response box. For example, if they saw “3 6 2 9,” they had to type 4730 into the box. For the *add3* problems, participants had to add three to each digit and type their answer into the response box, such that the correct response to “3 6 2 9” would be 6952. Participants were told that we were “interested in how quickly people perform simple and challenging working memory tasks” and were instructed to type in their answers as quickly as possible, without any reference to accuracy. The total number of choices made as well as the number of *add1* and *add3* choices was recorded.

### Results and Discussion

**FIGURE 2:**
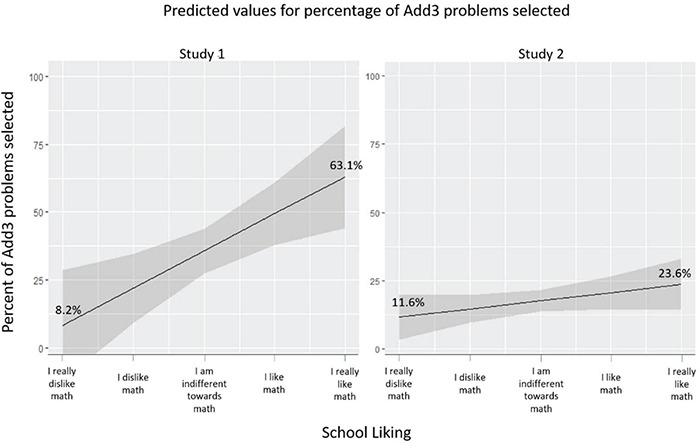
Predicted means for Studies 1 and 2.

On average, participants selected the *add3* task on 37% of trials (SD = 35.5%) and *add1* on the other 63%. A one-sample *t*-test showed that the average of *add3* trials was lower than the midpoint (50%), *t*(63) = −2.91, *p* = 0.005, supporting the previous assertion that people generally try to minimize effort by selecting the easier option. Table 1 reports the means, *SD*s, and correlations of all study variables. A multiple regression was conducted to examine the effects of math interest and math self-efficacy (both entered simultaneously as predictors) on the proportion of difficult problems (i.e., *add3*) selected. Interest predicted the proportion of difficult problems *(b* = 0.14, *SE* = 0.05, 95% CI [0.05, 0.23], β = 0.46, *p* = 0.004, see Figure 2 for predicted means). In contrast, self-efficacy did not *(b* = −0.05, *SE* = 0.06, 95% CI [−0.17, 0.08], β = −0.11, *p* = 0.468). Together, the two variables explained 16% of the variance (*F*(2, 60) = 5.65, *p* = 0.006).

**TABLE 1:** Descriptive statistics and correlations for Study 1.

	***M***	***SD***	**1**	**2**	**3**
1. Interest	3.10	1.19	–		
2. Self-efficacy	3.06	0.88	0.63**	–	
3. Proportion *add3*	0.37	0.36	0.39**	0.18	–
4. Fatigue	5.84	2.50	−0.40**	−0.30*	−0.29*

*p < 0.05, **p < 0.001.

To further capitalize on the nested nature of the data (especially given that our sample size of 63 is low for regression analyses), we repeated the analysis in a multilevel framework (using HLM version 7; Scientific Software International, 2000) with each choice (*N* = 5068; between 27 and 119 choices per participant) dichotomized as 0 = chose *add1*, 1 = chose *add3* as the level-1 dependent variable, and math interest and self-efficacy (uncentered) as level-2 predictors in a Bernoulli equation. Results indicate that interest was significantly related to a greater likelihood of selecting an *add3* problem (*OR* = 2.98, 95% CI [1.30, 6.83], *t*(60) = 2.64, *p* = 0.011), while self-efficacy was not (*OR* = 1.45, 95% CI [0.47, 4.46], *t*(60) = 0.80, *p* = 0.510)^6^.

Finally, to examine the effects of math interest on self-reported mental fatigue, a two-step multiple regression was conducted with interest and self-efficacy entered first, and the proportion of *add3* entered in the second step. In the first step, interest was significantly negatively related with fatigue *(b* = −0.74, *SE* = 0.32, 95% CI [−1.37, −0.10], β = −0.35, *p* = 0.025); self-efficacy was not (*b* = −0.24, *SE* = 0.43, 95% CI [−1.11, 0.62], β = −0.09, *p* = 0.579); the two variables explained 17% of the variance (*F*(2, 60) = 5.97, *p* = 0.004). In the second step, the proportion of *add3* trials selected was unrelated to fatigue, *(b* = −1.13, *SE* = 0.90, 95% CI [−2.93, 0.67], β = −0.16, *p* = 0.214). The effects of math interest diminished somewhat, *(b* = −0.58, *SE* = 0.34, 95% CI [−1.26, 0.10], β = 0.28, *p* = 0.094). Overall, the more a participant liked math, the less fatigue they experienced after engaging in the task, even after accounting for self-efficacy and the proportion of difficult trials (neither of which had an effect on fatigue).

As in previous research (Kool et al., 2010), we found that people are generally effort-aversive, with only 37% on average opting for the difficult choice. However, as expected, the results of this first study support our hypotheses that people will willingly choose to engage in more effort if the task is interesting. Participants who liked math were more likely to select more effortful math problems; those who disliked math were more likely to select the easier option. Additionally, math interest was related to experiencing less fatigue: despite engaging in more mentally effortful math problems for 15 min, those who generally enjoyed math reported less fatigue after the bout of effort. Math self-efficacy was unrelated to either the amount of effort or perception of fatigue, suggesting that the effects cannot be explained by feelings of self-efficacy derived from engaging in the math activity. That is, these results suggest that feelings of self-efficacy by themselves may not adequately explain why people would engage in an activity in the absence of tangible rewards, despite their correlation with interest.

## Study 2

The aim of this study was to replicate Study 1 with a larger sample and within-person measures of both the proportion of *add3* and fatigue. Since in Study 1 the question about math came at the end and may have been tainted by participants’ experience with the *add3* task, in Study 2 participants reported on this question before engaging in the *add1*/*add3* choice task. In this study, participants came into the lab to complete all measures on a computer. Instead of completing a single 15-min session of the *add1* versus *add3* task, participants completed four blocks of 5 min each; following each block, they completed measures of fatigue. Although we again expected to generally see some effort avoidance, we hypothesized that (1) math interest would predict a greater proportion of difficult (*add3*) problems selected; (2) math interest would predict less fatigue (despite solving more difficult problems).

### Participants and Procedure

We aimed to recruit as many participants as we could during a semester. Participants were 164 undergraduate students at the University of Toronto Scarborough, who completed the study for course credit. Participants first completed a packet of questionnaires (using Qualtrics) that included the same items as in Study 1 to assess math interest and self-efficacy (see OSF for full list of measures). They then completed the *add1*/*add3* choice task, which was the same as in Study 1, but with the following modifications: first, participants who completed fewer than three correct practice trials of either the *add1* or *add3* trials were asked to complete additional practice trials. Second, the choice task was presented in four blocks of 5 min each. After each block, participants were asked to report on feelings of fatigue using the following item: “I feel mentally exhausted right now,” rated on a scale of 1 (*strongly disagree*) to 7 (*strongly agree*)^7^. At the end of the four blocks, participants completed additional online questionnaires assessing affect and their perceptions of the task. Five participants were removed due to technical problems (results from choice task did not record). A further seven participants were excluded because of other problems with their data, resulting in a final sample of 152^8^.

### Results

On average, participants selected the *add3* task on 17.1% of trials (*SD* = 24.5). Eighteen percent of participants did not select the *add3* task on any trials, and a further 17% selected it on only one trial; this variable was thus highly skewed toward 0 (more on this below). Table 2 reports all the descriptive statistics. As in Study 1, math interest and self-efficacy were highly correlated, *r* = 0.63, *p* < 0.001. Neither math interest nor math self-efficacy were significantly correlated with average proportion of *add3* selected (i.e., how frequently they selected the add3 problems, *r* = 0.13, *p* = 0.10 for interest, *r* = 0.06, *p* = 0.438 for self-efficacy) or with average fatigue across the four blocks (*r* = −0.14, *p* = 0.095 for interest, *r* = 0.02, *p* = 0.831 for self-efficacy). To examine the proportion of variance that was due to between-person differences (i.e., how one person is different from another person) and within-person differences (i.e., how one block is different from other blocks for the same person), we computed the intraclass correlation (ICC) for the block-level variables (proportion of *add3*, fatigue). Only 17% of the variability in the proportions of *add3* selected was within-person (i.e., the reciprocal of the ICC), suggesting that this variable was predominantly a person-level difference: some people generally did more *add3* problems than others did. In contrast, fatigue was much more variable from block to block, with 66% of the variance at the within-person level.

**TABLE 2:** Descriptive statistics for Study 2.

	***M***	***SD***	**ICC**	**1**	**2**	**3**

**Between subject**						
1. Interest	2.80	1.28		–		
2. Self-efficacy	2.99	1.07		0.63**	–	

**Within subject**						

3. Proportion *add3*	0.17	0.26	0.83	0.13	0.06	–
4. Fatigue	4.75	1.77	0.34	–0.14	0.02	−0.09

Correlations for all variables are on the between-subject level. **p < 0.001.

We first tested our hypotheses that interest (i.e., liking math) is linked to selecting a larger proportion of more difficult problems. Given that the proportion of *add3* was non-normally distributed (skewness = 1.57; kurtosis = 1.71), and that no transformations fixed the skewness, there were a few different possible analytical strategies that could be used. We only realized after beginning data analyses that our initial planned analyses may not be adequate for this data (leading us to try different analyses), and different analyses gave slightly different results. Thus, we present all analyses here to allow the reader to draw their own conclusions. Given that we conducted analyses that deviated from our planned analyses, the following results should be considered exploratory and, thus, in need of independent confirmation. First, disregarding the non-normal distribution, we conducted a mixed analysis in SPSS with proportion of *add3* problems as the level-1 (block-level) dependent variable, the intercept specified as random, and math interest and self-efficacy as level-2 (person-level) predictors. Results showed that math interest was not a significant predictor of selecting more *add3* problems in each block *(b* = 0.03, 95% CI [−0.01, 0.07], *t* = 1.50, *p* = 0.136), and neither was math self-efficacy *(b* = −0.01, 95% CI [−0.06, 0.04], *t* = −0.33, *p* = 0.741).

Assuming that participants who chose either only one or none of the *add3* trials came from a different population (and that, since there was no variance to estimate, neither math interest nor math self-efficacy, nor any other variables, could account for it), we looked at only those participants who selected more than one *add3* problem (*N* = 99) and ran the same analyses as above. Results here showed that math interest was a significant predictor of selecting more *add3* problems in each block *(b* = 0.05, 95%CI [0.0004, 0.10], *t* = 2.00, *p* = 0.048), while math self-efficacy was not *(b* = −0.01, 95% CI [−0.07, 0.05], *t* = −0.20, *p* = 0.839). Next, looking at the total number of *add3* trials selected (on a person level, rather than within-person), we ran a negative binomial regression with math interest and self-efficacy specified as predictors, and the total number of trials attempted specified as the offset. Results showed that math interest was only marginally related to selecting more *add3* problems, *b* = 0.21, 95% CI [−0.02, 0.45], Wald χ^2^ = 3.18, *p* = 0.075, while math self-efficacy was unrelated, *b* = −0.056, 95% CI [−0.33, 0.22], Wald χ^2^ = 0.15, *p* = 0.695. Finally, we computed Kendall’s tau, a non-parametric correlation coefficient, assessing (separately) the links between interest and the proportion of add3 selected (tau = 0.04, *p* = 0.499), and between self-efficacy and the proportion of add3 (tau = 0.02, *p* = 0.756). Overall, across these different analyses, this study found mostly negative evidence for a relationship between math interest and the proportion of *add3* trials.

To test our second hypothesis regarding the effects of interest on fatigue, we again conducted a mixed analysis, this time with fatigue at the end of each block as the within-person dependent variable. Since fatigue was more normally distributed, we ran our initial planned analyses with the entire sample. Math interest was significantly related to less fatigue at each block *(b* = −24, 95% CI [−0.44, −0.04], *t* = −2.35, *p* = 0.02), while math self-efficacy was unrelated to fatigue *(b* = 0.20, 95% CI [−0.04, 0.45], *t* = 1.64, *p* = 0.102). The effects of math interest were stronger when only looking at participants who completed some *add3* problems (math interest *b* = −30, 95% CI [−0.55, −0.06], *t* = −2.42, *p* = 0.017), and also held when controlling for the number of *add3* problems completed in that block (see OSF for output from all analyses)^9^.

In this second study, we again tested the effects of math interest on choosing to exert more effort in a demand selection task, as well as on fatigue. The results for our first hypothesis (that math interest would predict the choice to exert more effort) were mixed: the effects were not significant when examining all participants, but were significant (albeit weak) when only looking at those who completed some *add3*. While in the first study participants selected the more difficult problems 36% of the time, this dropped to 17% in the second study, with 36% of participants selecting either zero or only one difficult (*add3*) problem. We are not sure what led to this difference between the two studies. Although they were some differences in the studies themselves (online vs. in-lab; conducted in December vs. January/February), there is no obvious reason why these differences should have influenced participants’ choices. However, the results for fatigue are more robust. In within-subject analyses, participants who liked math reported less fatigue after each block. This finding supports our hypothesis that people will be less fatigued following even a difficult task if they perceive it as enjoyable. This again suggests that interest can influence the phenomenology of exerting effort.

## Study 3

**FIGURE 3:**
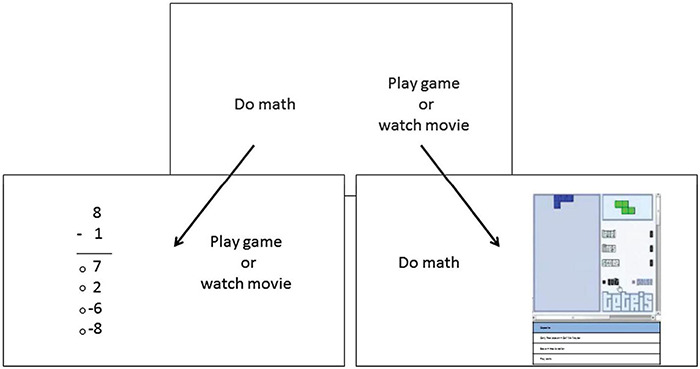
Graphical interface of the Academic Diligence Task (Galla et al., 2014). Participants toggle between solving math problems vs. playing Tetris or watching entertaining videos.

Study 3 replicates and extends results from Studies 1 and 2 using a different population of participants and different methods. In this field study involving a socioeconomically and racially diverse sample of high school seniors, participants completed a behavioral measure of self-control (Academic Diligence Task; Galla et al., 2014) in which they allocated their time between solving simple math problems (framed as beneficial for problem solving skills) and, alternatively, playing Tetris or watching entertaining videos (see Figure 3). Although Tetris requires cognitive effort, it contains elements that make it immediately rewarding/motivating (performance feedback, levels). Conversely, part of the effort required for the subtractions consists of forcing oneself to persist on a tedious task; this can feel more fatiguing than actually exerting cognitive effort (Milyavskaya et al., 2019). After the task, students rated the strength of temptation for Tetris and the videos. Students also completed self-report measures assessing school interest and general academic self-efficacy, mirroring the math liking and math competency questions used in Studies 1 and 2. Additionally, although this study did not assess feelings of fatigue, participants’ ratings of temptation strength allowed us to test whether interest was related to fewer temptations (experiencing temptations has previously been linked to greater feeling of fatigue; Milyavskaya and Inzlicht, 2017; Galla et al., 2018). Despite differences in methodology, our hypotheses remained the same: (1) school interest would predict a greater amount of time spent solving tedious, but “good for you” math problems in the presence of temptation; (2) school interest would predict less temptation for Tetris and entertaining video clips (despite exerting more effort from working longer on the math problems).

### Participants and Procedure

The final analytic sample included *N* = 447 high school seniors (*M*_age_ = 17.91, *SD* = 0.52) from a public high school in the northeastern United States. These participants were drawn from a larger study of 513 high school seniors. The final analytic sample reflects the number of participants who completed the Academic Diligence Task (see section “Measures” below). There was no overall stopping rule for data collection; the sample size reflects the maximum number of participants we were able to recruit within the allotted time provided by the schools. According to school records, 39% of the final sample were Black, 37% were White, 21% were Asian, and 3% were Hispanic; 54% were female. Just over half of participants (51%) were from low-income households, as indicated by their qualification for free or reduced-price lunch. In January of their senior year, participants completed a battery of measures that included the Academic Diligence Task and self-report questionnaires assessing school interest and self-efficacy during regular school hours on school computers. Students completed the self-report questionnaires before the Academic Diligence Task. Portions of the data from this study have been published elsewhere (Galla and Duckworth, 2015; Meindl et al., 2019), but the analyses reported here are novel.

### Measures

#### School Interest

Students answered three items (α = 0.83) about how interesting or enjoyable school is for them (“I like schoolwork,” “I find working on school assignments interesting,” “I like school more than most of my other activities”), from 1 = *strongly disagree* to 6 = *strongly agree* (*M* = 3.33, *SD* = 1.10).

#### Academic Self-Efficacy

Students also answered three items (α = 0.86) about their expectations to do well in school (“I know I can learn the material in my classes,” “I believe I can be successful in my classes,” “I am confident that I can understand the material in my classes”), from 1 = *not at all true* to 5 = *completely true* (*M* = 4.14, *SD* = 0.73).

#### Academic Diligence Task

This is an online task that involves a split-screen interface with the choice to either complete single-digit subtraction problems (“Do Math”) or to play Tetris or watch video clips (“Play game or watch movie”; for more details about the task, see Galla et al., 2014). In this shortened version, following a 30-s practice block of math problems, the main task involved three, 3-min blocks during which participants were free to toggle between completing the skill-building activity or passing the time by playing Tetris or watching videos. To make doing the math problems seem worthwhile, participants read a story that emphasized the utility of completing subtraction problems. Specifically, participants read the following prompt: “New scientific research shows that students who practiced math by doing more subtraction problems went on to earn higher grades. Even doing simple and easy math problems can make you a better problem solver, which can help you in all areas of your life.” They also read that whenever they felt like it they were free to play Tetris or watch videos, but that the more problems they solved the more their problem-solving abilities would improve. To be consistent with Study 1 and 2, we used the total percentage of time students spent on the math skill-building exercise, averaged across all three blocks, as a measure of task engagement^10^.

Following the 9-min task, students rated their perceptions of how tempting Tetris or the videos were, from 1 = *not at all tempting* to 5 = *very tempting* (*M* = 2.95, *SD* = 1.31).

#### Demographic Covariates

Due to significant (*p* < 0.05) differences across demographic subgroups on key predictors and outcomes, students’ gender, race/ethnicity, and eligibility for free or reduced-price lunch were included as covariates in models reported below (see OSF for results of these demographic comparisons).

### Results and Discussion

**FIGURE 4:**
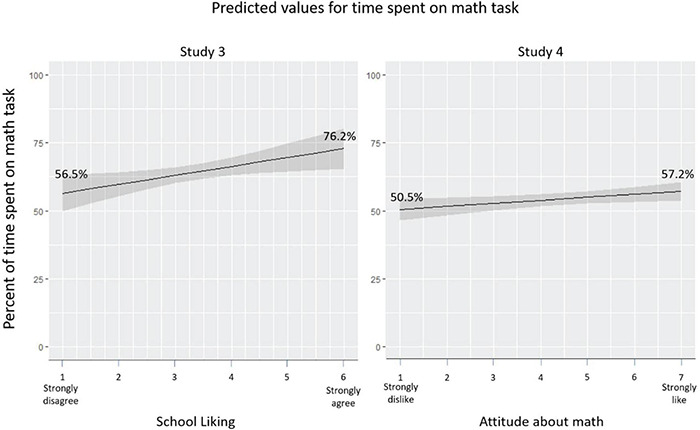
Predicted percent of time spent on math task in Studies 3 and 4.

On average, participants spent 64% of the time on the math skill-building exercise (*SD* = 0.30). This is perhaps not surprising given the rationale presented to students for completing the math problems; the predicted means across levels of interest are shown in Figure 4. Across the course of the study, students tended to reduce their engagement with the math task: Participants spent 71% of the time on the skill-building exercise during the first 3-min block, 64% during block 2, and only 57% during block 3, a significant linear decrease, *b* = −0.07, 95% CI [−0.09, −0.05], *p* < 0.001.

School interest and academic self-efficacy were positively correlated, *r*(445) = 0.25, *p* < 0.001, but the magnitude of the relation between them strongly suggests they were separate factors. Students with strong interest in school spent more time on the math skill-building portion of the Academic Diligence Task, *r*(445) = 0.14, *p* = 0.004. Likewise, students who reported feeling capable of doing well in school (i.e., higher self-efficacy) also spent more time on the math skill-building exercise, *r*(445) = 0.09, *p* = 0.049. School interest, *r*(445) = −0.18, *p* < 0.001, but not self-efficacy, *r*(445) = −0.06, *p* = 0.203, was significantly negatively correlated with the strength of temptation for Tetris or the videos.

Mixed linear regression analysis (MIXED command in SPSS) corroborated these results. In this analysis, percent time spent on math skill-building exercise was the level-1 time-varying dependent variable, and school interest and self-efficacy were grand-mean-centered level-2 predictors. School interest, *b* = 0.03, 95% CI [0.01, 0.06], *p* = 0.013, but not academic self-efficacy, *b* = 0.03, 95% CI [−0.01, 0.06], *p* = 0.192, predicted more time spent on the math skill-building activity. Adding demographic covariates to the model did not change the estimates substantially, but now both school interest, *b* = 0.03, 95% CI [0.01, 0.06], *p* = 0.008, and academic self-efficacy, *b* = 0.04, 95% CI [0.004, 0.08], *p* = 0.031, predicted more time spent on the math skill-building activity.

Multiple regression analysis, with video temptation as the dependent variable, revealed a similar pattern of results. School interest, *b* = −0.21, 95% CI [−0.32, −0.10], *p* < 0.001, but not academic self-efficacy, *b* = −0.03, 95% CI [−0.20, 0.14], *p* = 0.733, predicted less temptation toward Tetris or the videos during completion of the math skill-building activity. Adding covariates did not shift the estimates substantially: interest continued to predict less temptation, *b* = −0.21, 95% CI [−0.33, −0.10], *p* < 0.001, whereas self-efficacy did not, *b* = −0.06, 95% CI [−0.23, 0.11], *p* = 0.458. Even when controlling for the average time spent on the math skill-building exercise (and demographics), school interest still predicted less temptation, *b* = −0.16, 95% CI [−0.26, −0.05], *p* = 0.004.

Conceptually replicating results of Studies 1 and 2, students who enjoyed school spent more time engaging on a tedious academic exercise in the presence of tempting diversions (but not solving more problems; see Footnote 9). Students who enjoyed school also experienced less temptation for the diversions, even though they engaged in more mental effort. Although this study did not directly examine fatigue, previous research has found that people report feeling more depleted when they experience greater temptation (Milyavskaya and Inzlicht, 2017). The next study, however, does examine fatigue directly. Additionally, in this study, interest and self-efficacy were not math-specific, but for school more broadly. Previous research, however, suggests that academic self-concept is differentiated based on subject matter (Marsh and Shavelson, 1985; Green et al., 2007), so that interest and self-efficacy are better examined separately for different subjects (i.e., math vs. English or science). This is remedied in Study 4.

## Study 4

Study 4 provided a conceptual replication and extension of Study 3. In this study, high school seniors completed a longer version of the Academic Diligence Task (five, 4-min blocks) and a single item measure assessing math interest. As in Study 2, students provided ratings of mental fatigue after each of the task blocks.

### Participants and Procedure

Participants were *N* = 921 high school seniors (*M*_age_ = 17.90, *SD* = 0.51) from two public high schools in the Northeastern United States. These participants were drawn from a larger study on college persistence. As in Study 3, we recruited as many participants as we could within the allotted time provided by the schools. According to school records, 36% of participants were Black, 33% were White, 21% were Asian, and 8% were Hispanic, and 2% were of other or mixed ethnic backgrounds; 49% were female; and 55% qualified for free or reduced-price lunch. Participants completed a battery of measures that included the Academic Diligence Task and a self-report questionnaire assessing math interest during regular school hours on school computers. Students at one high school completed the self-report questionnaire before the Academic Diligence Task; those at the other school completed the survey in a separate session some months after the Academic Diligence Task. Portions of the data from this study have been published elsewhere (Galla et al., 2014), but the analyses reported here are novel.

### Measures

#### Math Interest

Students’ liking of math was assessed using a single item, “Please rate your attitude toward math,” that was rated from 1 = *strongly dislike* to 7 = *strongly like*. Single-item measures of attitudes have been shown to demonstrate equivalent predictive validity to multiple-item measures of the same attitude (Bergkvist and Rossiter, 2007). Data on this measure were available for 884 of the 921 students (*M* = 4.39, *SD* = 2.14, range = 1 to 7).

#### Academic Diligence Task

In this longer version, students first completed a 60-s practice block of math problems and then completed five, 4-min blocks during which they were free to toggle between completing the math activity or pass the time by engaging with Tetris or the videos. Participants read a story similar to that described in Study 3 to provide a rationale for students to spend time solving the math problems. As in Study 3, we used the total proportion of time students spent on the math skill-building exercise, averaged across all five blocks, as a measure of task engagement^11^.

After each task block, students rated how tiring it was to do the math (1 = *not at all tiring* to 5 = *very tiring*). This repeatedly assessed item served as our measure of mental fatigue. Overall fatigue, averaged across all five blocks, was *M* = 2.84, *SD* = 1.04, range = 1 to 5.

#### Demographic Covariates

Due to significant (*p* < 0.05) differences across demographic subgroups on our key predictors and outcomes, students’ gender, race/ethnicity, and eligibility for free or reduced-price lunch were included as covariates in models reported below (see OSF for results of demographic comparisons). We also included a dummy code for school affiliation, since students were drawn from two different high schools.

### Results and Discussion

On average, participants spent 54% of the time on the math skill-building exercise (*SD* = 0.33). As in Study 3, participants reduced the time spent on the math problems throughout the task: Participants spent 70% of the time on the skill-building exercise during the first block and only 44% during the fifth block, a significant linear decrease, *b* = −0.06, 95% CI [−0.06, −0.05], *p* < 0.001. Also consistent with Studies 1 and 3, bivariate correlations showed that math interest was positively associated with cumulative time on task during the Academic Diligence Task, *r*(882) = 0.07, *p* = 0.031. In contrast, math interest was negatively correlated with cumulative mental fatigue, *r*(882) = −0.16, *p* < 0.001. Thus, students who liked math exerted more effort on a tedious math skill-building activity (forgoing entertaining videos and games), yet simultaneously reported less overall mental fatigue. Interestingly, cumulative mental fatigue and time on task (averaged across task blocks at the between-person level) were uncorrelated, *r*(919) = 0.03, *p* = 0.344. However, multilevel models showed that fatigue and time on task were tightly coupled at the within-person level, such that students who spent more time on task in a given block reported higher fatigue, *b* = 0.16, 95% CI [0.04, 0.28], *p* = 0.010.

Mixed linear regression analysis corroborated these results. In this analysis, grand-mean-centered level-2 math interest significantly predicted level-1 time-varying time on task, *b* = 0.01, 95% CI [0.001, 0.02], *p* = 0.031. Adding level-2 covariates of school attended, gender, race/ethnicity, and socioeconomic status did not change the estimate for math interest, *b* = 0.01, 95% CI [0.005, 0.03], *p* = 0.005. This suggests that students who liked math were more likely to increase their time spent solving math problems following prior exertion of the same math skill-building activity.

We fit a similar multilevel model, but used level-2 math interest to prospectively predict level-1 time-varying fatigue rather than time on task. Controlling for the effects of demographic characteristics, math interest predicted less fatigue on the subsequent block, *b* = −0.07, 95% CI [−0.11, −0.04], *p* < 0.001. This suggests that students who like math were less likely to experience increases in fatigue following the exertion of effort on a math skill-building activity.

## Mini Meta-Analysis of All Results

As our studies had somewhat different measures of our variables of interest and yielded slightly different (although mostly converging) results, we conducted a series of mini meta-analyses to synthesize the results of all studies (Goh et al., 2016). We were especially interested in testing the relationships between both interest and ability (controlling for the other) with willingness to expend effort and perceptions of fatigue; we thus used the Pearson correlation (*r*) as our effect size and computed partial correlation coefficients between the relevant variables in each study. Although two of our studies (Studies 2 and 4) contained within-subject data, we were unable to convert the output into an appropriate effect size, so looked at the between-subject correlations. Finally, the studies did not contain measures of all the same key constructs. While all four studies contained measures of interest and engagement in effortful task (proportion of *add3* selected in Studies 1 and 2; percentage of time spent on math vs. Tetris in Studies 3 and 4), only Studies 1, 2, and 4 had measures of fatigue, and Study 4 did not contain a measure of self-efficacy (beliefs of being good at math in Studies 1 and 2, academic self-efficacy in Study 3). Our mini meta-analyses thus contain different numbers of studies depending on the effect of interest. All the meta-analyses use a fixed-effects approach.

We conducted a total of four mini meta-analyses (see Table 3). First, we used the partial correlation coefficient from all four studies for the correlation between variables representing interest and engagement in more effortful tasks (controlling for self-efficacy in Studies 1-3), weighted *r* = 0.107, 95% CI [0.06, 0.16], *z* = 4.22, *p* < 0.001, suggesting a small but reliable positive effect of interest on choosing to engage in more cognitive effort. Second, we used data from Studies 1, 2, and 3 to compute partial correlations between self-efficacy and effortful engagement. The overall weighted *r* for this analysis was *r* = 0.064, 95% CI [−0.012;0.140], *z* = 1.66 which was not significantly different from 0 (*p* = 0.10). Note, however, that this was also not significantly different from the effect of interest (*z* = 0.93, *p* = 0.35). The third mini meta-analysis examined the relation between interest and fatigue (Studies 1, 2, and 4), finding a small but reliable effect whereby people who experienced more interest felt less fatigued after performing a difficult task *(r* = −0.169, 95% CI [−0.226; −0.111], *z* = 5.66, *p* < 0.001). Finally, combining two studies (1 and 2) where we had self-efficacy and fatigue showed that the effect size of self-efficacy on feelings of fatigue was small and not reliable *(r* = −0.100, 95% CI [−0.230; 0.033], *z* = −1.48, *p* = 0.14). Table 3 reports all the raw effects sizes (*r*s) and sample sizes used in each study, as well as the results from the meta analyses.

**TABLE 3:** Internal mini meta-analysis of four studies.

	**Meta 1: Interest and choice**	**Meta 2: Self-efficacy and choice**	**Meta 3: Interest and fatigue**	**Meta 4: Self-efficacy and fatigue**
Study 1 (*N* = 63)	0.362	–0.094	–0.285	–0.070
Study 2 (*N* = 158)	0.114	0.043	–0.182	–0.112
Study 3 (*N* = 447)	0.136	0.093		
Study 4 (*N* = 884)	0.073		–0.159	
Weighted *R*	0.107**	0.064	−0.169**	–0.100
*z*	4.22	1.66	–5.66	–1.48

**p < 0.001.

## General Discussion

In this paper, we examined interest and self-efficacy as predictors of effort and fatigue, for the purpose of generating novel hypotheses about behavior and phenomenology. We initially expected that enjoyment/interest would predict (1) choosing to engage in relatively more mental effort when provided with limited incentives to do so; and (2) experiencing the effort as less fatiguing. For the first hypothesis, the individual results from each of the four studies were somewhat mixed. However, a meta-analysis of the results across the four studies suggests that there was a small *(r* = 0.11) yet robust (*z* = 4.22, *p* < 0.001) overall effect of interest on engagement in mental effort. We thus cautiously conclude that interest does lead people to willingly exert greater effort. However, more research is needed to provide stronger evidence and identify boundary conditions and moderators. On the other hand, we found consistently strong support for our second hypothesis concerning the phenomenology of exerting mental effort. In all studies that included measures of fatigue (Studies 1, 2, and 4), math interest predicted lower feelings of fatigue, even after engaging in more mental effort. Importantly, these effects were found across studies from two different research programs, in two different tasks, with different populations. Thus, while the meta-analytic effect size was modest *(r* = −0.17), the reliability of the relationship suggests that interest may diminish the perceived fatigue of effortful tasks.

The present research has implications for how we understand self-control, fatigue, and effort, and raises further questions about how effort is evaluated. In line with prior research suggesting that effort is aversive (e.g., Kool et al., 2010), we found that people avoided cognitive effort in a laboratory demand selection task (Studies 1 and 2) and reduced effort over time in a real-world task (Studies 3 and 4). Recent valuation-based models of self-control (Berkman et al., 2017; Shenhav et al., 2017; Galla et al., 2018) provide a rational and mechanistic account of mental effort, where exerting effort comes with costs that are balanced with rewards obtained for that effort. But when people choose to exert more effort due to interest, is it because effort is less costly (since the person does not feel like they are really putting in effort) or more rewarding? In other words, interest may not actually reduce the cost of effort, but may increase the value of the effortful option; our paradigm did not distinguish between these possibilities. Future research is needed to further examine the mechanism by which interest influences people’s decisions to engage in effort and effort perception.

Our research also follows a recent focus on the phenomenology of effort and control. While some have proposed that cognitive control is aversive and accompanied by negative affect in the absence of reward (e.g., Kool et al., 2010; Saunders et al., 2015), our research suggests that this may not always be the case. That is, we found that when participants are interested in and enjoy an objectively effortful task, they do not feel fatigue to the same extent as participants who do not enjoy the task. This phenomenology, in turn, is what might drive behavior—and particularly decisions to engage in further control—which supports previous findings that it is the perception of having exerted control that may matter more than the actual effort/control (Clarkson et al., 2010; Job et al., 2010).

People who enjoy a given task may be more likely to perceive that engaging in the task is easier or less tiring, which in turn facilitates further task engagement. As seen in the results from Study 3, this association may also occur because people perceive alternate activities or options as less tempting. If we assume that self-control involves “overcoming a temptation or prepotent response in favor of a competing goal” (Milyavskaya et al., 2018), and that applying self-control is effortful and aversive (Inzlicht et al., 2015), then it follows that if the temptation is lessened, then less self-control is actually needed—resulting in less effort and less fatigue, yet greater performance. The present studies, however, were unable to test this pathway directly (i.e., whether perceiving the task as easier/less fatiguing led to greater behavior engagement), because phenomenology was measured after the behavior (that is, after participants decided whether or not to engage in further control). Future research is needed to properly disentangle the directionality of these effects to better understand the relationship between interest, phenomenology, and behavior. Additionally, while the present study only examined feelings of fatigue, future research can further examine the effects of interest on other aspects of phenomenology, such as negative affect more generally as well as positive affect and flow.

In addition to examining effects of interest on effort, we also examined the possible effects of self-efficacy. Given that previous research has found interest and self-efficacy to be strongly related, we wanted to ensure that any effects of interest were not merely due to self-efficacy. In the present studies, we again found that self-efficacy was related to interest, *r*s ranging from 0.25 to 0.65, such that people who considered themselves better at math also liked it more. However, in our studies, only interest, and not self-efficacy, reliably predicted both greater behavioral persistence and lower feelings of fatigue (although the differences between the effects of self-efficacy and interest were not significantly different). This suggests that effects of self-efficacy on persistence found in previous studies (e.g., Liem et al., 2008) may have been due to the role of the shared variance with interest—that is, efficacy may lead to greater persistence because tasks where one feels efficacious are perceived as more enjoyable. Alternatively, other experiences akin to feelings of competence or self-efficacy – for example engaging in an activity to diagnose self-efficacy, or to prove to themselves or to others that they are indeed capable – may also lead to persistence in effortful activities. Experimental and longitudinal research needs to further examine the relationship between interest, self-efficacy, and effort to better understand the directionality of the effects (but see Lent et al., 2008, for research suggesting that over long periods of time self-efficacy affects interest but not vice versa). In the short term, it may be that self-efficacy leads to interest, but it also may be that interest leads to effort and persistence, which then builds self-efficacy.

Much recent research on mental self-control has relied on artificial laboratory tasks such as judging numbers based on color or parity (Kool et al., 2010), or in our case the *add1*/*add3* task. Conversely, most real-world self-control problems are not true drudgery, so it is unclear whether the results from these laboratory studies generalize into real-world tasks. The Academic Diligence Task used in Studies 3 and 4 of the present paper (see also Galla et al., 2014) presents a more ecologically valid scenario, at least for students who are often caught between the necessity of engaging in effortful and/or tedious cognitive work and the tempting pull of watching TV or browsing social media. In this task, we did not see effort avoidance, but instead more time spent on the effortful task (64% in Study 3, 54% in Study 4). Although there were no direct rewards in the task to engage in the effortful schoolwork (students were only reminded of the long-term value of practicing math skills for their problem-solving abilities), it may be that students have learned to value schoolwork and have internalized it as instrumental value or learned industriousness (see Inzlicht et al., 2018). It may also be the case, however, that the school setting in which the study took place resulted in a perceived situational demand or expectation that time be spent on schoolwork. Future research should endeavor to bring research on cognitive control into the real world, to examine the self-control choices that people make in real time throughout their day and link that to cognitive control (for an example, see Powell et al., 2017).

Following most research on cognitive control, the present research operationalized effort as engagement in an objectively more difficult task. While it may be reasonable to assume that working on a more difficult task is more effortful, this operationalization does not lend itself to obtaining an exact measure of how much actual effort was exerted by each participant, nor participants’ direct perceptions of this effort. This distinction between objective and subjective effort is only now beginning to emerge in the literature on physical effort. For example, Marcora et al. (2009) have shown that participants cycling at 80% of their maximum output (i.e., same objective level of effort) perceived this effort differently depending on whether they had previously been exposed to a mental fatigue manipulation. That is, in some instances, putting in the same amount of physical effort *feels* more effortful. In studies on cognitive effort, however, the exact amount of effort cannot be directly measured or manipulated, and proxy measures of engagement in difficult tasks are used.

In addition to its relevance to research and theories on cognitive control, our research also raises some questions for self-determination theory (Deci and Ryan, 2000). Specifically, in our studies, the construct of interest paralleled self-determination theory’s conceptualization of intrinsic motivation. However, in self-determination theory research, intrinsic motivation is often combined with identified and integrated motivation (engaging in an activity because of personal importance or “fit” with the self) to form a measure of autonomous motivation (Sheldon and Elliot, 1999; Milyavskaya et al., 2014). It is unclear whether and how intrinsic motivation translates into the other components of autonomous motivation (identified and integrated). Future research can further look at the differences between these different components of autonomous motivation, and see whether they differentially impact perceptions of effort and willingness to exert effort. For example, in Studies 3 and 4, the Academic Diligence Task was presented as being good/valuable for participants, which is similar to the definition of identified motivation; participants chose to engage with this task over 50% of the time. It would thus be interesting to examine when interest and value have similar or different effects on willingness to exert effort. For example, as people feel more tired, value might matter less, but interest may play more of a role.

### Limitations

One limitation of the present research is our measurement of interest and self-efficacy. In Studies 1, 2, and 4 we only used one item, which assessed how much participants liked math (for interest), or how good they were at math (for self-efficacy). Although liking most closely connotes intrinsic motivation and interest, it may have assessed something slightly different (e.g., enjoyment, positive affect, etc.; Iran-Nejad, 1987). However, using “liking” as a measure of interest is common in the literature (e.g., Tanaka and Murayama, 2014). By teasing out self-efficacy in our analyses, we can be more confident that liking math is not due to positive feelings stemming from feelings of competence/self-efficacy. Additionally, both interest and self-efficacy were assessed for math in general, rather than for the specific task at hand (adding or subtracting); in Study 3, they were even more abstract (for schoolwork in general). These general feelings may have only partially translated into task-specific interest and self-efficacy, which may account for the small effect sizes observed in this research. For self-efficacy, since the tasks were relatively simple (e.g., simple subtraction), even low general math self-efficacy may have translated into high feelings of self-efficacy on the problems. Perhaps self-efficacy would have played a greater role for a more difficult task. Future research could use a better measure with more varied items to better assess the relevant constructs. Assessing them for the task itself (rather than the general domain as was done in this study) may also show a stronger relation.

Related to the issue of measurement of interest and self-efficacy was our design of the tasks. In Studies 1 and 2, the task was designed specifically to equate the two choices on everything except required effort (so that add3 is effortful, but not more or less inherently enjoyable, than add1). In Studies 3 and 4, the academic diligence task offered participants the choice of doing simple math or playing Tetris/watching videos. The latter options were not equivalent in the amount of effort required (as Tetris would undoubtedly require more cognitive effort than videos), and there is no clear reason to expect than doing simple math problems would require more cognitive effort than playing Tetris. This aspect of the design, that the alternative to math may have been equally effortful, does not detract from our point, however – Tetris is inherently enjoyable, which would lead participants to select it even it required as much effort as math; interest in math, however, may balance out the inherent interest of Tetris, pulling participants to do more math problems. Unfortunately, the task did not provide a breakdown of whether participants chose to do Tetris or watch videos when they were not doing math.

Another limitation of the present research is that participants were limited to high school and university students. As such, we do not know whether the results would generalize to other age groups. It would be especially interesting to examine this phenomenon with older adults. In Studies 3 and 4, the sample was diverse in terms of race/ethnicity and SES, but there were some demographic differences (i.e., by gender, race/ethnicity, and SES) that related to our variables of interest; the generalizability of the effects might thus depend on the composition of future samples. It is also important to note that the overall effect sizes between interest and mental effort and fatigue were small by conventional standards. Our use of tedious activities, as opposed to more demanding ones, may have blunted the association between interest in math and effort. Though this is not a limitation *per se*, a prediction for future research is that interest will be more tightly coupled to effort and fatigue on tasks that activate and challenge valued skill sets.

### Conclusion

Overall, this research found a small but consistent effect of interest on the willingness to exert effort and on reduced perceptions of fatigue. The focus of this paper stands out among most other research on cognitive control, which has emphasized the cost and disutility of mental effort. The present research serves as a reminder that it is important to not overgeneralize—although mental effort is costly, it is not uniformly so. We need to ask ourselves not only for whom is it costly, but also in which situations. We found that interest can be powerful in overcoming the cost of mental effort both for choosing to engage in greater effort and for feelings of fatigue, despite a lack of any overt rewards. The effects were robust (albeit small) across various studies.

## Data Availability Statement

The datasets for Studies 1 and 2 can be found in online repositories. Data from Studies 3 and 4 were collected from minors, and we do not have consent to publicly post those data (for questions or requests regarding this data, please contact the second author). The names of the repository/repositories and accession number(s) can be found below: https://osf.io/sn376.

## Ethics Statement

The studies involving human participants were reviewed and approved by University of Toronto REB (Studies 1–2) and University of Pennsylvania IRB (Studies 3–4). For studies 3 and 4, although participants were minors. Written informed consent from the participants’ legal guardian/next of kin was not required to participate in this study in accordance with the national legislation and the institutional requirements.

## Author Contributions

MM and MI designed the first two studies in the manuscript. MM conducted and analyzed those two studies and wrote up the methods and results, conducted the meta analysis, and wrote the manuscript with input from BMG, MI, and ALD. BMG and ALD designed and conducted studies 3 and 4 in the manuscript. BMG analyzed and wrote up those studies. All authors contributed to the article and approved the submitted version.

## Conflict of Interest

The authors declare that the research was conducted in the absence of any commercial or financial relationships that could be construed as a potential conflict of interest.

## Publisher’s Note

All claims expressed in this article are solely those of the authors and do not necessarily represent those of their affiliated organizations, or those of the publisher, the editors and the reviewers. Any product that may be evaluated in this article, or claim that may be made by its manufacturer, is not guaranteed or endorsed by the publisher.

## Footnotes

1 We had initially predicted that interest, rather than self-efficacy, would lead to more effort (i.e., choosing effortful, rather than easier, behaviors), and constructed our hypotheses accordingly, conducting confirmatory analyses (as described in each individual study). However, after a more thorough review of the literature, we recognized that there is no particularly compelling reason to think that interest would be more important than self-efficacy, as there is some evidence that each could contribute to effort.

2 We did not initially predict main effects for self-efficacy, but did plan to include it in the analyses as a control variable to rule out alternate possible explanations for engaging in greater effortful tasks and reduced feelings of fatigue.

3 https://osf.io/sn376/.

4 We had opened the study to allow for 100 sign-ups (based on a power analysis, to detect a correlation coefficient of 0.3 with 80% power, 84 participants would have been necessary); 17 students signed up on the participant pool, but did not begin the study.

5 This study also tried to manipulate interest/importance for the task by having participants write about how they use their working memory in pleasurable activities in their daily life (interest condition), or asking them to practice their working memory by writing in detail about the route they took to get to school. However, based on the responses people gave, the manipulation did not seem to elicit interest (e.g., “working memory is used to remember phone numbers heard”). There was no effect of the manipulation on any outcome variables.

6 Results from a unit-specific model with adaptive Gaussian quadrature are reported here; the entire output file is available at https://osf.io/sn376.

7 Participants also answered items on energy (“I feel energized right now”) and boredom (“I feel bored right now”) using the same 7-point scale. These were included to examine potential overlap or differences between these affective states, but were only weakly correlated with fatigue (*r*s = 0.11 to 0.21 for fatigue and boredom, *r*s = −0.04 to −0.21 for fatigue and energy), and so were not considered further.

8 In the data file, there is a code of “problem” for these participants, but we could not recall the reason for it, and cannot find anything in our files that could help us figure it out, so cannot recreate the reasons for exclusion. However, the results are the same when they are included; these results are posted on https://osf.io/sn376.

9 We also tested the interaction between the number of *add3* problems and interest on fatigue; the interaction term was not significant (*b* = 0.46, 95%CI [−0.20, 1.12], *t* = 1.37, *p* = 0.172).

10 To promote transparency, we note that Academic Diligence Task data reported in Study 3 have been published elsewhere (Galla and Duckworth, 2015). In that study, we operationalized self-control (or, diligence) as an average of z-score standardized time on task (reported here) and the number of problems solved correctly (*M* = 144, *SD* = 83, *r* = 0.89). Results using number of problems solved correctly and the composite score are reported online (https://osf.io/sn376). In these analyses, we find that self-efficacy is a statistically significant predictor of number of problems solved correctly and the composite diligence score (including covariates), whereas intrinsic value of school is not. However, intrinsic value in school remains a significant predictor of the strength of temptation for the videos or Tetris, regardless of whether we control for time on task, number of problems solved correctly, or the composite diligence score.

11 Similar to Study 3, we also collected total number of problems solved correctly in each task block. Substituting number of correctly solved problems did not substantively alter conclusions reported in Study 4. For details, see https://osf.io/sn376.
